# Supplementary material for: An information-theoretic approach to single cell sequencing analysis
Source: BMC Bioinformatics. 2023 Aug 12;24:311. doi: 10.1186/s12859-023-05424-8 (PMC10422744; doi:10.1186/s12859-023-05424-8)
Supplement: Supplementary file 1 — Additional file 1. Two additional figures and one additional table: Expression of Hbb-bs in the Stumpf data set (Supplementary Figure 1); Feature selection benchmarking of scEC and scEC-impute (Supplementary Figure 2); and Contingency table between cell annotations provided in the Stumpf data set and scEC labels. [file 12859_2023_5424_MOESM1_ESM.pdf]

Supplementary Figure 1

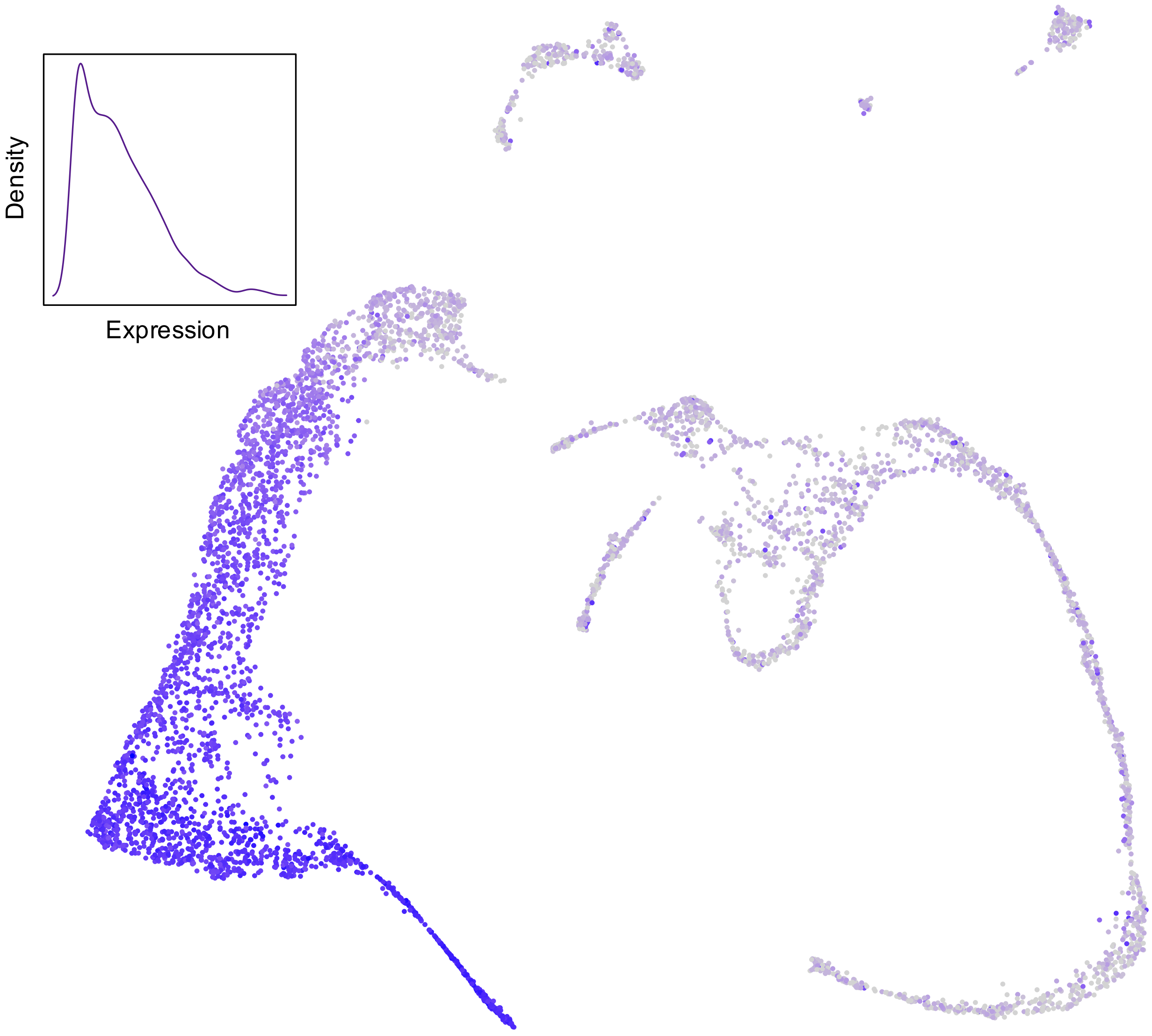

Expression of *Hbb-bs* in Stumpf et al. (2020) data set. *Hbb-bs* is identified by information-theoretic feature selection – based on $I(g)$ – but not by `scran` (Lun et al. 2016); *Hbb-bs* has a complex distribution, with at least two modes of expression in erythroblasts (inset shows probability density, y-axis, of transcript counts, x-axis, in erythrocyte lineage), with a high level of expression in mature erythrocytes and some non-zero level of (possible technically-induced) expression across less developed erythroblasts and other cell types. The non-parametric nature of $I(g)$ means it can robustly identify informative genes, even in the presence of multimodal expression patterns.

Supplementary Figure 2

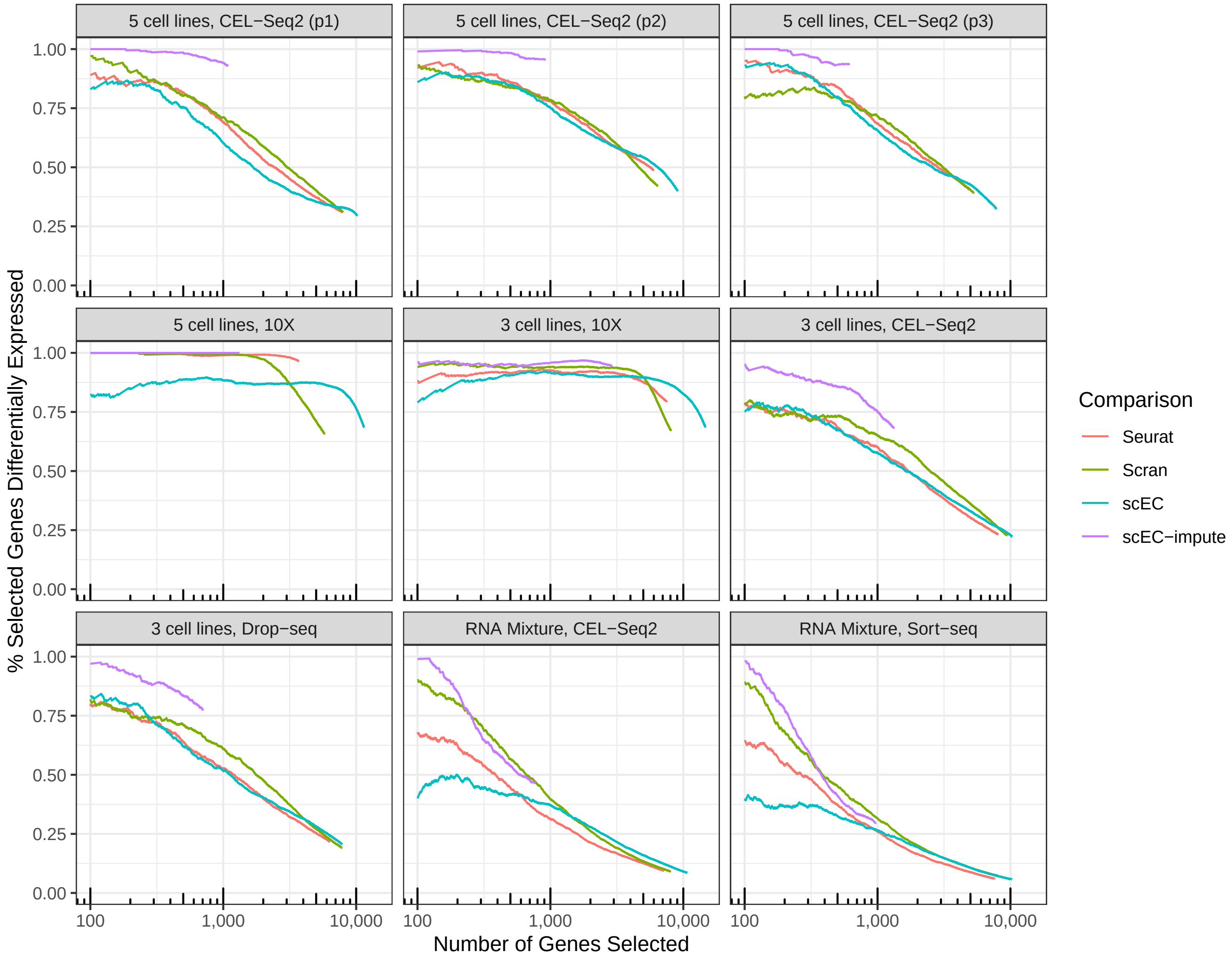

Feature selection benchmarking of scEC and scEC-impute. he percent of the top N genes by different feature selection metrics that are differentially expressed. Data is Sc-seq from three or five cancerous cell lines, or a mixture of RNA from said cell lines, sequenced by specified platform (Tian et al. 2019). In each case, scEC-impute has the greatest fidelity in selecting differentially expressed genes without access to cluster labels, with the exception of the RNA mixture data sets where it draws equal with the other methods after several hundred genes have been selected. Number of differentially expressed genes identified in each study by Wilcox test of each cell line against remaining, false discovery rate corrected p-value < 0.05: 5 cell lines, CEL-Seq2 (p1) 3695; 5 cell lines, CEL-Seq2 (p2) 4523; 5 cell lines, CEL-Seq2 (p3) 3390; 5 cell lines, 10X 7851; 3 cell lines, 10X 10195; 3 cell lines, CEL-Seq2 2712; 3 cell lines, Drop-seq 2005; RNA Mixture, CEL-Seq2 947; RNA Mixture, Sort-seq 667.

Supplementary Table

Erythroblasts	2436	171	0	0	0	1	0	0	1	0	233	0	3	0
Myeloblasts	39	4	313	0	0	0	0	0	0	0	0	245	2	0
Pro-B	38	0	0	187	0	0	0	0	0	0	0	0	0	0
Pre-B	0	0	0	58	0	0	0	0	2	0	0	2	0	0
Monocytes	0	0	0	23	165	0	0	0	4	2	0	23	0	0
Basophils	0	0	0	0	0	130	0	0	0	0	2	1	0	0
Pericytes	0	0	0	0	0	0	110	0	0	2	0	0	0	0
Megakaryocytes	0	0	0	0	0	0	0	40	0	0	21	0	0	0
T-NK	3	0	0	0	0	0	0	0	56	0	0	1	0	0
Endothelial Cells	0	0	0	0	1	0	4	0	0	41	4	0	0	0
HSPCs	49	4	3	5	1	7	0	0	2	0	182	50	0	0
Monoblasts	7	0	16	7	66	0	1	0	0	0	3	200	0	0
Neutrophils	0	0	0	1	1	1	0	0	1	0	0	8	255	0
Myelocytes	2	0	4	3	0	0	0	0	0	0	0	93	164	0

Contingency table between cell annotations provided in (Stumpf et al. 2020) and scEC labels. Note that while the number of clusters for scEC was fixed at 14, only 13 clusters were realised; generally, the number of clusters returned will match the number specified, except in cases where any further splits in clusters provide only a minor increase in inter-cluster heterogeneity, so are harder to find by numerical optimisation.
